# Cost analysis of day care centres in Norway

**DOI:** 10.1371/journal.pone.0219568

**Published:** 2019-08-08

**Authors:** C. Vossius, G. Selbæk, J. Šaltytė Benth, A. Wimo, K. Engedal, Ø. Kirkevold, A. M. M. Rokstad

**Affiliations:** 1 Centre for Age-Related Medicine, Stavanger University Hospital, Norway; 2 Research Centre for Age-related Functional Decline and Disease, Innlandet Hospital Trust, Brumunddal, Norway; 3 Norwegian National Advisory Unit on Ageing and Health, Vestfold Hospital Trust, Tønsberg, Norway; 4 Institute of Clinical Medicine, University of Oslo, Oslo, Norway; 5 Department of Geriatric Medicine, Oslo University Hospital, Oslo, Norway; 6 Health Services Research Unit, Akershus University Hospital, Nordbyhagen, Norway; 7 Division of Neurogeriatrics, Department of Neurobiology, Care Sciences and Society (NVS), Karolinska Institutet, Stockholm, Sweden; 8 Centre for Research & Development, Uppsala University/County Council of Gävleborg, Gävle, Sweden; 9 Norwegian University of Science and Technology (NTNU) Department of Health Sciences in Gjøvik, Gjøvik, Norway; 10 Faculty of Health Sciences and Social Care, Molde University College, Molde, Norway; University of Malaya, MALAYSIA

## Abstract

**Background:**

Day care services aim to offer meaningful activities and a safe environment for the attendees and a respite for family caregivers while being cost effective. This study compares the use of formal and informal care in users and non-users of day care centres designed for persons with dementia.

**Method:**

Users of day care designed for dementia (DC group) and non-users (NDC group) were followed over a period of 24 months or until nursing home admission (NHA) respectively death. Demographic and clinical characteristics were collected at baseline and after 12 and 24 months. The use of care was recorded by Resource Utilization in Dementia (RUD).

**Results:**

A total of 257 persons with dementia participated in the study, 181 in the DC group and 76 in the NDC group. Users of day care centres cause higher costs due to the expenses for day care, while neither the use of home nursing, secondary care, informal care nor the time until NHA did show any differences between users and non-users. The overall costs were higher in the DC group at baseline and after 12 months, but this difference was no longer present at the end of the two-year study period.

**Conclusion:**

Our results indicate no potential cost-saving effect of day care designed for people with dementia, as the use of day care did neither result in a reduced use of care nor in a delay of NHA. Future research should balance the non-monetary benefits of day care against its costs for a full cost-effectiveness analysis, most favourable in a RCT-design.

## Introduction

During the course of their disease persons with dementia require an increasing amount of care due to their declining mental and physical health. Specially designed day care services offer care and supervision during day time in order to reduce the carer burden for family carers while at the same time offering meaningful activities and a safe environment for persons with dementia in order to maintain function in activities of daily living and improve the quality of life of the users. Additionally, it is suggested that such services may delay the need for nursing home placement and thus be cost effective [1–7].

In Norway, health and care services including home care, day care and nursing homes are organized by the municipalities, who also bear the main share of costs, while out-of-pocket contributions by the users are relatively small. Some of these services like providing home care and nursing home placement for those regarded in need are mandatory services, while other services like day care centres are optional, and their provision is decided by the municipality’s council. As a measure to enable persons with dementia to remain home-dwelling as long as possible especially designed day care services was one of the priorities in the first governmental Norwegian Dementia Plan 2015 [8], and this commitment has been maintained in the second Dementia Plan, 2020, aiming at an establishment of such centres in every municipality by 2020 [9,10].

However, there is only limited information concerning the health economic effects of day care centre programmes designed for people with dementia in Norway. Repeated reviews made by the Norwegian Knowledge Centre for the Health Service (NOKC) [11,12] identified only eight published studies of moderate to poor quality. At societal level, two of these studies reported lower costs for the day care centre group, due to reduced admittance to acute hospitals or nursing homes [10,7]. Still, cost effectiveness could not be confirmed [13].

The project «Effects and costs of a day care centre program designed for people with dementia–a 24-month controlled study» (ECOD) includes 257 persons with dementia of which 181were users of day care centres and 76 were not [14]. Recent findings suggest that the use of day care centres did not result in fewer nursing home admissions (NHAs) [15]. The aim of the present study is a cost analysis that compares the use of formal care in the primary and secondary health sector, and the use of informal care rendered by next of kin in users and non-users of day care centres included in the ECOD project.

## Methods

### Study design and setting

The ECOD study followed two groups of persons with dementia: i) a group of users of day care in centres designed for persons with dementia (DC group), and ii) a group that did not use day care designed for persons with dementia (NDC group). Both groups were followed until NHA or death, respectively, or, if none of these events occurred, over a period of 24 months. It was considered unethical to deprive participants from visits at a day care centre. Hence, the study was not design as a randomized controlled study but as a quasi-experimental trial. Participants in the NDC group were only recruited in municipalities that did not offer day care designed for persons with dementia, but might offer other forms of day care like stay in a senior centre, or day care in a nursing home open for old and frail people, while day care specially designed for persons with dementia includes activities according to the individual person’s preferences and abilities to take part. Participants were identified via local authority dementia teams and in-home care service offices. Participants in the DC group were only recruited in the municipalities that offered day care designed for persons with dementia. Any type of day care centre with a program designed for people with dementia was eligible for inclusion in the study, and participants were identified via the participating day care centres. The participants in both groups were recruited from urban and rural areas including all regions of Norway.

### Participants

The following inclusion and exclusion criteria were used: participants should be 65 years or older, have a dementia diagnosis according to the ICD-10 criteria [16], a Mini Mental State Examination (MMSE) score of ≥ 15 [17], and the capacity to give informed consent based on the judgement of a professional caregiver. Additionally, there should be a family caregiver willing to participate. Potential participants having applied for permanent nursing home placement or suffering from a serious co-morbid physical disorder with a life expectancy less than six months were excluded. Participants in the DC group should have attended the day care centre for at least four weeks but no longer than twelve months and visit the centre at least twice a week. Inclusion period lasted from December 2013 to July 2015.

### Data collection

Data was collected at baseline (BL), at the first follow-up after 12 months (FU1) and at the second follow-up after 24 months (FU2) after BL examination by a group of 13 trained assessors including nurses, occupational therapists and a psychologist. As demographic data age, gender and whether the participant was living alone was collected. Clinical data collected is described below, while the participants’ use of formal and informal care was assessed by the tool Resource Utilization in Dementia (RUD) [18].

The diagnosis of dementia at BL was set according to the criteria of ICD-10 [15]. The diagnosis of dementia was set independently by two specialists in psychiatry and experienced in old age psychiatry and research, based on all available information about the participants. Discrepancies in diagnoses were settled in a consensus meeting.

The Resource Utilization in Dementia (RUD) assesses the use of the patient’s formal and informal care during the last four weeks and is answered by a close family carer [18].

The Clinical Dementia Rating Scale (CDR) was applied to assess the severity of dementia [19]. The rating scale comprises six items. For statistical purposes we calculated the CDR-sum of boxes (CDR-SoB) that offers an extended range of values and is calculated by adding the item scores(range 0–18), where higher scores indicate more severe dementia [20].

The Neuropsychiatric Inventory nursing home version (NPI-NH) assesses neuropsychiatric symptoms. The instrument contains 12 items and is conducted as an interview with a carer Severity (scored 0–3) was multiplied by frequency (scored 0–4), giving an item score from 0–12, where higher scores indicate more severe symptoms [21].

The Physical Self-Maintenance Scale (PSMS) consists of six items (scored 1–5) and assesses personal activities of daily living (PADL) function. The overall score ranges from 6 to 30, where higher scores indicate higher PADL dependency [22].

The Instrumental ADL scale (IADL) scale assesses the instrumental activities of daily living and has eight items. Maximum score ranges from 8 to 31, and higher scores indicate higher IADL dependency [22].

The General Medical Health Rating (GMHR) rates physical health. It consists of one item, with the four categories excellent, good, fair or poor [23].

The Relatives’ Stress Scale (RSS) assesses the caregivers’ level of stress related to caregiver burden. The RSS consists of 15 questions with five alternative answers with the scoring range from 0 (never) to 4 (very often/always), giving a minimum score of 0 and a maximum score of 60, with a higher score indicating more severe stress [24].

### Costs for health care services and informal care

The use of the following health care services was assessed: home nursing, home help, day care centre, in-hospital stay, visits at out-patient clinics, and visits at the emergency room. Costs for the use of formal health care services including in-hospital care were calculated by applying the unit prices stated in the report “Resource Use and Disease Course in Dementia” [25]. As these unit prices are established for the year 2013, we inflated the costs to 2017 prices according to the consumer price index (CPI) as stated by Statistics Norway [26]. All prices are expressed in Euro (1 € = 9.53 NOK; 8.8.18) The following prices were applied: home nursing 73 € per hour; home help 59 € per hour; day care centre 100 € per day; in-hospital stay 1453 € per day; out-patient clinic 138 € per visit; emergency room 234 € per visit. Costs were subsumed as “Costs for primary care” including home nursing, home help and day care, and as “Costs for secondary care” including in-hospital stays, visits at out-patient clinics, and visits at the emergency room.

As the observation period was determined as 24 months or until NHA or death, respectively, the use of nursing home care was not included into the study protocol. To calculate the difference in the use–and hence the costs—of nursing home care between the two cohorts, we established the time until NHA, defined as time under observation from study start to NHA.

Informal care included help by next of kin as well as the wider network consisting of other family members or friends. Costs for informal care are calculated as follows: Mean wages in Norway were 28.83 € per hour in 2017 [27]. For participants where the primary carer was in paid work, costs were calculated at the hourly rate of 28.83 €. When the primary carer was not in paid work, the hourly rate for leisure time was applied, being 35% of the mean wages or 10.09 € [27]. As we had no information about the working status of the wider social network, and the contribution of the network only represented about 3% of the total amount of informal help, the same costs were applied for help rendered by others than the primary carer.

Costs are calculated from a societal point of view, comprising expenses borne by the public as well as out-of-pocket contributions.

### Statistical analysis

Missing values in the different instruments were imputed on the item level for cases with at least 50% of items available. Random numbers drawn from an empirical distribution generated for each item of interest were used to substitute missing values.

Demographic and clinical characteristics were presented as means and standard deviations (SD), or frequencies and percentages, as appropriate. Independent samples t-test and χ^2^-test were used for comparison of demographic and clinical characteristics in DC and NDC groups at baseline.

The differences between the DC and NDC group were assessed by linear mixed models. Differences in secondary care received were assessed by a generalized linear model with dichotomous variable as outcome (secondary care vs. no secondary care), as the distribution was extremely skewed with 84% of patients with zero secondary care costs. Random effects for users nested within day centres were included into all models. Fixed effects for non-linear time, group variable and the interaction between the two were entered. Further, the models were adjusted for user characteristics measured at baseline (age, gender, living alone, and GMHR) or simultaneously with clinical variables (CDR-SoB, NPI, PADL, IADL, RSS). Unadjusted (no adjustment variables) and adjusted models including all characteristics listed above were estimated. Adjusted models were further reduced by applying Akaike’s Information Criterion (AIC), however only the generalized linear model was possible to reduce. Results of unadjusted models were illustrated graphically, while results of adjusted models were tabulated as regression coefficients and standard errors or odds ratio (OR) and 95% confidence intervals (CI). P-values for group comparison at each time point as well as changes in time within groups were derived from the models in a post hoc analyses.

To assess differences in mean observation time in nursing home care stratified by reason of leaving the study, a linear mixed model with fixed effects for group, reason and the interaction between the two was estimated. Random effects for centre were included.

All statistical analyses were performed in SPSS v 25 or SAS v 9.4. Results with p-values below 0.05 were considered statistically significant.

### Ethical consideration

The project has been approved by the Regional Committee in Ethics in Medical Research in South-East Norway (2013/1020). After written and oral information, the patients and the family caregivers were asked to give written informed consent. Only patients with the capacity to give consent were included.

## Results

### Participants

A total of 257 persons with dementia participated in the study, 181 in the DC group and 76 in NDC group. In the DC group 47 participants completed FU2 (26.0%), while 93 (51.4%) had been admitted to nursing homes, 15 (8.3%) had died and 26 (14.4%) had dropped out due to withdrawal or other reasons. In the NDC group 16 (21.1%) completed FU2, while 35 (46.1%) had been admitted to nursing homes, 5 (6.6%) had died and 20 (26.3%) had dropped out due to withdrawal or other reasons. Demographic and clinical data at BL for the study population as a whole and for the separate groups are presented in Table 1. Participants in the NDC group were more frequently female, had poorer somatic health, less severe dementia, less severe neuropsychiatric symptoms, lower IADL dependency and the next of kin presented lower levels of stress than participants in DC group.

**Table 1:** Demographic and clinical characteristics at BL for the whole study population and according to whether the participant was in the DC or NDC group.

	All participants (n = 257)	DC group (n = 181)	NDC group (n = 76)	p-value
Age, mean (SD)	81.5 (6.4)	81.1 (6.5)	82.4 (6.0)	0.146^1^
Gender, female (%)	168 (65.4)	110 (60.8)	58 (76.3)	0.017^2^
Living alone (%)	135 (52.5)	92 (50.8)	43 (56.6)	0.400^2^
GMHR poor or fair (%)	54 (22.5)	28 (17.0)	26 (34.7)	0.002^2^
CDR-SoB, mean (SD)	6.4 (2.5)	6.8 (2.5)	5.5 (2.3)	<0.001^1^
NPI, mean (SD)	5.9 (4.7)	6.3 (4.8)	5.0 (4.5)	0.037^1^
PSMS, mean (SD)	9.4 (3.1)	9.5 (3.2)	9.3 (3.1)	0.573^1^
IADL, mean (SD)	21.9 (5.3)	22.6 (5.1)	20.4 (5.6)	0.003^1^
RSS, mean (SD)	17.7 (11.0)	19.1 (11.1)	14.1 (9.8)	0.001^1^

BL = baseline; DC = users of day care centres designed for dementia; NDC = non-users of day care centres designed for dementia; SD = Standard deviation; GMHR = General medical health rating; CDR-SoB = Clinical dementia rating scale–sum of boxes; NPI = Neuropsychiatric inventory; PSMS = Physical self-maintenance scale; IADL = Instrumental activities of daily living; RSS = Relatives’ Stress Scale.

^1^ Independent samples t-test;

^2^ χ^2^-test

### Use of formal and informal care and costs

#### Primary care

**Fig 1:**
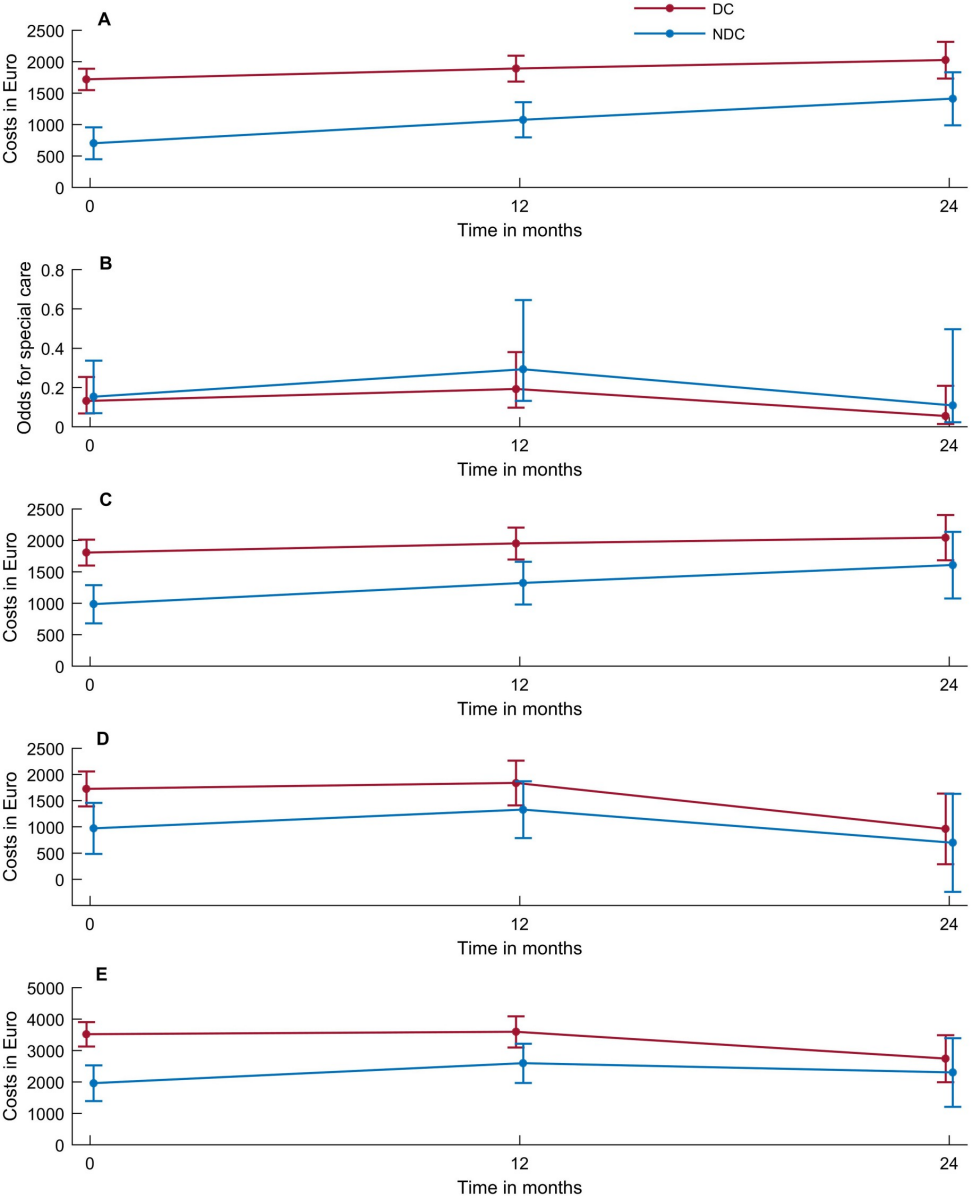
Time trends for the DC group and NDC group at BL, FU1 and FU2. ***A***: *Cost for primary care;* ***B***: *Odds for secondary care;* ***C***: *Cost for formal care;* ***D***: *Costs for informal care;* ***E***: *Total costs*. BL = baseline; FU1 = follow-up1 at 12 months; FU2 = follow-up2 at 24 months; DC = users of day care centres designed for dementia; NDC = non-users of day care centres designed for dementia.

The use of home nursing, home help and day care centres as well as the overall costs for primary care are presented in Table 2. According to linear mixed model, primary care costs were significantly higher for participants in the DC group in the unadjusted model (p<0.001 at BL and FU1, p = 0.019 at FU2, Fig 1A) and in the adjusted model (p<0.001 at BL and FU1, p = 0.007 at FU2.

**Table 2:** The use of primary, secondary and informal care and costs in the month prior to BL, FU1 and FU2, respectively.

	BL			FU1			FU2		
	DC group (n = 181)	NDC group (n = 76)	p-value	DC group (n = 103)	NDC group (n = 40)	p-value	DC group (n = 47)	NDC group (n = 16)	p-value
Visits at day care centre, mean (SD)	11.4 (4.7)	4.6 (1.9)	<0.001	11.7 (6.4)	1.0 (2.5)	<0.001	12.0 (5.8)	3.1 (4.4)	<0.001
Home nursing, hours, mean (SD)	7.5 (13.3)	7.2 (8.9)	0.976	6.8 (10.3)	10.6 (9.6)	0.255	9.0 (12.6)	12.3 (15.9)	0.149
Home help, hours last month, mean (SD)	0.6 (1.3)	1.7 (2.8)	<0.001	0.6 (1.1)	2.0 (2.6)	<0.001	0.8 (1.5)	2.4 (4.1)	<0.001
Cost for primary care, mean (SD)	1729 (1166)	718 (781)	<0.001	1698 (1028)	1037 (893)	<0.001	1840 (1282)	1360 (1531)	0.019
Days in hospital, mean (SD)	0.06 (0.35)	0.20 (0.86)	0.064	0.04 (0.32)	0.08 (0.47)	0.230	0.11 (0.73)	0.19 (0.75)	0.828
Visits at out-patient clinic, mean (SD)	0.09 (0.33)	0.13 (0.41)	0.917	0.28 (1.08)	0.25 (0.54)	0.137	0.20 (0.93)	0.75 (3.00)	0.107
Visits at emergency room, mean (SD)	0.07 (0.28)	0.03 (0.16)	0.171	0.05 (0.27)	0.05 (0.22)	0.637	0	0	-
Secondary care, yes (%)	28 (15)	12 (16)	0.708	17 (17)	11 (28)	0.288	4 (9)	2 (13)	0.364
Costs for formal care, mean (SD)	1820 (1337)	1010 (1547)	<0.001	1809 (1101)	1192 (893)	0.001	1942 (1517)	1736 (1951)	0.173
Informal help, hours, mean (SD)	113 (162)	63 (114)	0.003	143 (187)	58 (72)	0.012	69 (87)	61 (85)	0.228
Costs for informal care, mean (SD)	1806 (2601)	1056 (1386)	0.005	2020 (3174)	849 (837)	0.051	907 (1621)	1272 (1835)	0.595
Total costs, mean (SD)	3616 (2926)	2068 (2056)	<0.001	3704 (3356)	2021 (1323)	0.002	2696 (1984)	3008 (2796)	0.463

BL = baseline; FU1 = follow-up1; FU2 = follow-up 2; SD = Standard deviation; DC = users of day care centres designed for dementia; NDC = non-users of day care centres designed for dementia. Costs in Euro, applying 2017 prices. Primary care includes day care centre, home nursing and home help. Secondary care includes in-hospital stay and visits at out-patient clinic and emergency room. Formal care includes primary and secondary care. Total costs include costs for formal and informal care.

P-values derived from linear mixed model for continuous and generalized linear model for dichotomous variables, adjusting for correlations within persons nested within centres.

#### Secondary care

In-hospital stays, visits to the out-patient clinic, visits to the emergency room and secondary care received are presented in Table 2. According to the generalized linear mixed model, there were no differences between the two groups in the secondary care received in the unadjusted (Fig 1B) or adjusted model.

#### Time to NHA

Mean time under observation during the study period was 458 days for the DC group and 449 days for the NDC group. According to the linear mixed model, the mean observation time was not different between the two groups, when stratified by “completed FU2” (p = 0.997), “NHA” (p = 0.256), “death” (p = 0.270), or “drop out” (p = 0.859). Consequently, there were no differences in costs for nursing home care between the DC and NDC group.

#### Costs for formal care

Costs for formal care are presented in Table 2 and Fig 1C. According to the unadjusted and the adjusted model (Table 3) costs for formal care were significantly higher for participants in the DC group at BL (p<0.001) and FU1 (p = 0.001) but not at FU2.

**Table 3:** Results of linear and generalized linear mixed models.

Variable	Costs for formal care (primary and secondary care)		Costs for informal care		Total costs (formal and informal care)	
	Regr.coeff. (SE)	p-value	Regr.coeff. (SE)	p-value	Regr.coeff. (SE)	p-value
Intercept	1571.72 (909.42)	0.086	1904.64 (1497.59)	0.205	3207.05 (1687.54)	0.059
Time	10.54 (21.90)	0.631	38.11 (41.95)	0.365	47.39 (48.34)	0.328
Time x Time	0.07 (0.89)	0.940	-2.53 (1.71)	0.140	-2.14 (2.00)	0.285
Group NDC–ref.	0		0		0	
DC	749.19 (174.58)	**<0.001**	533.90 (301.53)	0.077	1374.90 (331.44)	**<0.001**
Time x Group	-15.13 (14.15)	0.287	-24.02 (26.40)	0.364	-52.17 (27.14)	0.057
Age	-30.35 (11.43)	**0.009**	-32.66 (18.89)	0.085	-59.58 (21.25)	**0.006**
Gender						
Female–ref.	0		0		0	
Male	-379.11 (165.16)	**0.023**	202.80 (270.95)	0.455	-178.57 (305.70)	0.560
Living alone						
No–ref.	0		0		0	
Yes	833.28 (161.23)	**<0.001**	5.23 (264.91)	0.984	913.91 (298.98)	**0.003**
GMHR						
Poor or fair	-245.38 (168.67)		459.08 (278.51)	0.101	304.97 (313.63)	0.332
Good or excellent–ref.	0	0.148	0		0	
CDR SOB	-8.17 (34.64)	0.814	126.27 (60.02)	**0.036**	125.62 (67.73)	0.065
NPI	-5.47 (15.13)	0.718	59.22 (25.80)	**0.022**	55.79 (29.04)	0.056
PADL	98.00 (24.71)	**<0.001**	14.57 (42.90)	0.734	100.16 (47.54)	**0.036**
IADL	37.49 (16.46)	**0.023**	25.06 (28.41)	0.378	55.82 (32.09)	0.083
RSS	-0.12 (6.96)	0.986	-7.09 (11.80)	0.548	-3.62 (13.22)	0.784

SD = Standard deviation; DC = users of day care centres designed for dementia; NDC = non-users of day care centres designed for dementia. GMHR = General medical health rating; CDR-SOB = Clinical dementia rating scale–sum of boxes; NPI = Neuropsychiatric inventory; PSMS = Physical self-maintenance scale; IADL = Instrumental activities of daily living; RSS = Relatives’ Stress Scale.

Costs represent costs per month.

#### Informal care

The hours and costs of informal care during the month prior to BL, FU1 and FU2 are presented in Table 2. In the unadjusted model participants in the DC group received significantly more informal help at baseline (p = 0.003) and T12 (p = 0.012), while the differences were not statistically significant in the adjusted model (numbers not presented).

Costs for informal care are presented in Table 2 and Fig 1D. In the unadjusted model, there was a significant difference between the DC and NDC group at BL (p = 0.005) while there was no difference in the adjusted model (Table 3).

#### Total cost

Total costs are presented in Table 2 and Fig 1E. According to the unadjusted and the adjusted model (Table 3) total costs were significantly higher for participants in the DC group at BL (p<0.001) and FU1 (p = 0.002 in unadjusted and p = 0.020 in adjusted model) but not at FU2. There were no significant changes over time in neither the DC nor the NDC group.

## Discussion

This study represents a comparison of the use and costs of care between users and non-users of day care centres designed for persons with dementia. Our findings indicate that users of day care centres cause higher costs to society due to the expenses for day care, while neither the use of home nursing, secondary care, informal care nor the time until NHA did show any significant differences between users and non-users. Over the course of two years the overall costs were higher for participants in the DC group at BL and FU1, but this difference was no longer present at the end of the two-year study period.

Day care centres have received an increased attention in the recent years, as a measure to prolong time until NHA for persons with dementia. Nursing home stay is by far the most expensive primary care intervention for persons with dementia in the public health care sector^25^, and any intervention resulting in a delay of NHA will probably free economic and personnel resources that will be needed to meet an increasing number of elderlies in the coming decades. Wimo et al found in a Swedish study over one year that a cohort of day care users were less frequently admitted to nursing home [7], however, cost-effectiveness could not be established [13]. In the ECOD study we could not find any delay in NHA. A previous analysis of the same material even showed a higher probability for NHA in users of day care centres [14]. Engedal found in a previous Norwegian study performed thirty years ago that the use of day care centre was associated with a reduced frequency in acute hospital admission and hence lower costs, but could not find a delay in NHA [10]. A review published in 2004 reports that day care might be cost effective, and “that the benefits of day care might be similar or greater than those achieved through standard care [28] while a Swedish report concludes: “There is insufficient scientific evidence for the cost-effectiveness of dementia programs and environmental interventions, such as day care (…)” [29]. In the present study the use of day care centres seems to add on to the overall costs of care for persons with dementia and is not compensated by a reduced use of formal or informal care. However, the differences in costs between the two groups were most significant at BL and no longer present at FU2. This might be partly due to a selection bias, where participants with a higher need for care dropped out due to death or NHA. Unfortunately, the statistical power towards the end of the study period was too low to allow for a thorough analysis of this topic.

The pricing of informal care is discussed in the literature as it describes assumed costs for care where no actual payments are made [30,31]. We chose to price informal care as suggested by Johannessen et al [32]. However, different pricing models for informal care would not have altered the overall conclusion in our findings.

### Strengths and limitations

The strengths of this study is the inclusion of participants from a high number of day care centres in a large geographical area including rural as well as urban locations all over Norway and an observation period of two years. We applied standardised test and evaluation instruments that have been widely used in previous research about persons with dementia. The assessment tools have been tested for reliability and validity in Norwegian studies.

However, the design of the study has some major limitations. Due to ethical considerations we could not chose a randomised controlled trial (RCT) design, as this would have deprived some of the participants of attending a day care centre for the duration of the study period. Therefore, we chose to carry out a quasi-experimental trial by including participants in the NDC group exclusively in municipalities that did not offer day care designed for persons with dementia. We might thus have introduced a selection bias, as the NDC group contained participants that did not have the option to attend specially designed day care, while the DC group exclusively comprised participants who chose to attend while leaving out those who chose not to attend of reasons not captured by this study. Additionally, we were not able to recruit 200 participants in each group, as was originally aimed for [14], and with 76 versus 181 participants the NDC group was significantly smaller. Further, there was a higher drop-out rate than expected during the observation period, thus reducing statistical power and the significance of the results and weakening the generalizability of our finding to the population of persons with mild to moderate dementia.

For a full economic evaluation of day care centres, participants should be followed from a defined point of time—like first-time diagnosis—and until death. While participants in the DC group were included one to twelve months after they had started in a day care centre, participants in the NDC group were included at an even less defined point of time. The interval from the first symptoms or first-time diagnosis of dementia to study inclusion was not recorded, and information about the time of survival after NHA was not collected. The observation period of two years might as well be too short to cover the effect on costs over time, as we observed changes in the difference between the two groups during the study period. We were thus not able to evaluate the full effect of day care on the overall care costs of persons with dementia. However, the direction of the results might indicate no potential cost-saving effect of day care designed for people with dementia.

## Conclusion

Our results indicate no potential cost-saving effect of day care designed for people with dementia, as the use of day care did not result in a reduced use of formal or informal care, and neither in a delay of NHA. However, the effect on the patients’ and proxies’ health and quality of life as outcome measures was not explored. Future research should balance the non-monetary benefits of day care against its costs for a full cost-effectiveness analysis, most favourable in a RCT-design.
